# On the Conduct of Medical Societies

**Published:** 1887-08

**Authors:** D. R. Wallace


### Society Notes.

### ON THE CONDUCT OF MEDICAL SOCIETIES.

*A Paper Read before the Terrell Medical Society, by Dr. D. R. Wallace, August 1, 1887, and printed at the special request of said Society members.*

Contributed to Daniel's Texas Medical Journal by vote of the Society.

It is not a pleasant reflection—I am almost unable to bring myself to realize it—but the facts are becoming so many, pointed and troublesome to explain upon any other hypothesis, I guess I shall have to give in and confess. *I am getting to be an old man.* One whose natal year was emphasized by the inauguration of John Quincy Adams as the sixth President of the United States; the recognition of the independence of Brazil by Portugal; the creation of the republic of Boliviar—Bolivar, the great South American Washington, having voluntarily, and against the wishes of a majority of his countrymen, resigned the dictatorship; the recognition of the independence of Hayti by France; the opening of the Erie Canal; the illumination of the City of New York by gas;—events which, in our time, begin to recede into the dim and shadowy past—I say, one whose natal year was marked by such events cannot boast of being a *very young man*. If I have succeeded in proving what you would have been quite willing to take my word for, corroborated by the evidence of your eyes, that I am an old man, I trust I may presume to claim one of the old man's privileges (he hasn't many), to-wit: that of giving advice to his juniors in regard to matters they most probably know much more about than he does. You all recollect the anecdote of the wife belaboring her husband, and when asked what she was doing, the husband replied for her: "Oh, let Betsy alone—*it does her a power of good, and does not hurt me a bit!*" Similarly, it does us old members of the profession a power of good to advise you younger ones, makes us feel like we are indeed Nestors for the time being, the repositories of the wisdom of three generations, *and does not hurt you a bit.*

I know it is much easier to preach than to practice; to give advice than follow it. There is that, also, about advice giving, that, being a modest man, I dislike very much—may say, is quite offensive to me. It is this: it carries along with it a sort of implied claim to superiority which, I beg to assure you, I am very far from feeling. It seems to say or to savor of something like this: "Listen to me; I am wiser than you; I know something you do not, but which you would be the wiser for knowing."

With these playful preliminaries, intended to disarm your criticism, and introduce more gracefully what is to follow, I proceed to the task I propose to myself—only observing, what you doubtless would find out without my calling your attention to it, that not the least effort will be made at fine writing, or to please the fancy by any display of rhetoric. I shall, in as few words and as plain language as I can command, place you in possession of some thoughts deemed worthy of your attention—mainly the suggestions of my own observations, and reflections upon the *best method of conducting a medical society so as to make the meetings result in most benefit to the membership.*

I need not detain you to insist that every member shall come prepared to say something, to contribute his share to the general interest. What that something is to be, should not be left to chance to determine, but should be the selection of quiet deliberation, as to what will be of most benefit to himself and others. Having made his selection, he should have a clear, clean cut conception, a definite picture of the matter before his mind's eye. Having such, he will find no trouble in expressing it clearly and in the fewest words. Vividness of conception suggests strong, pointed and expressive language.

It is related of the great Athenian general, Phocim, who, although not a professional orator, was no mean rival of Demosthenes, that he was observed on one occasion just before going to an assembly of the people, rapt in profound thought. Asked upon what so intently engaged, he replied, "I was trying to shorten my speech." His brief, caustic, sententious speeches were the dread of his great rival, and wrung from him the expression, upon seeing him come into the public assembly on an important occasion, "Here comes the cleaver of my harangues." How often have I thought of the words "*trying to shorten my speech*" since I read them in Plutarch nearly half a century ago. When listening to some wordy speaker, it would occur to me, "this good man has not taken time, or been at the pains, to shorten his speech. Wordiness arises from ignorance, or vagueness of conception, which is about the same thing. You will find old Socrates was right in saying "All men express themselves easily enough, and with all needy clearness, on matters in regard to which they are well informed." The degree of clearness of expression is the exact correlate of clearness of idea.

Where shall he find, how select his subject? Just here it occurs to me a great mistake is made, not only in medical societies but in other learned bodies as well. It is all too common to select subject couched in big high sounding words—to make a grand display—something to be talked about as an Eighth World's Wonder. Surrounded by encyclopedias and other learned tomes, it may not be difficult to display an extent of knowledge, and to marshall learned terms and rounded sentences to the great admiration of the *ignorant many*, but to the *thoughtful well-informed few*, such displays are generally simply words, sound, air in motion;—simply this and nothing more.

Were it not the wiser, more sensible course, for a member of society like yours to select some case from his case book, in the management of which he was at sea, some case in which the obscurity or contradictory character of the symptoms left him in doubt what course to pursue, or again some case in which the patient died, when he thought he should have gotten well,—all medical men meet such,—state his case fairly, give his treatment frankly, and learn if he can, by conference with his fellows, what course they would have pursued under the circumstances, and why his treatment failed;—all done in a common sense way and in few plain words. Be not afraid of exposing your ignorance;—sensible people never are, when they wish to learn. It is the fool that would make believe that he knows it all. Sensible people would rather seem to know less than they do.

Again you all read, or ought to, current medical literature. It is chaffy, you say, like Gratiano's reasons. "As two grains of wheat, hid in two bushels of chaff, you shall seek, all day ere you find them, and when you have them, they are not worth the search." All this is true enough ; yet medical journals are not more worthless than other periodicals. It is in this way the world moves,—ten thousand abortive efforts, for one short step of advance; ten thousand theories and speculations for one small truth. It is the duty of the medical man to the extent of his natural endowments to give his patrons the benefit of all that is known to the profession. This he can do in this age of material progress, of unprecedented advance in the development of the physical sciences that make up, or are auxilary to, the great science of medicine from which is derived the healing art, only by constant reading, intense study and close observation.

In order to make his reading most instructive, the reader should turn it over in his own mind and compare his conclusions with those of his fellows. It is to be hoped that there is no member of the Terrell Medical Society who reads so little, thinks so little of what he reads, or who meets so little in his daily work, at the bedside and elsewhere, that puzzles him, as not to have some such matter, as above indicated, to bring to the attention of his fellows at every meeting. If there be such a one, he is too indolent or selfposing to be a doctor, and we may as well turn him over to Voltaire's definition of a medical man, as "one who pours drugs of which he knows little, into stomachs of which he knows less. The trouble is not the lack of such matter and cases, but a sort of habitual indifference of observation into which a class of medical men fall, than which there is nothing more fatally obstructive to professional progress. When a physician ceases to observe, he ceases to grow, and when he ceases to grow, he begins to die.

Right here I hazard an assertion,—one, some of you will hesitate to believe ; will receive, if at all, *cum grano salis.* It is this ;— If such member will come to every meeting of your society with some practical matter to submit, some practical enquiry to make, some item of practical information, gleaned from his reading or the result of observation, to relate—wasting no time in wordy declamation,—your society will soon grow into such interest, that the members will not only not willingly absent themselves from its meetings, but will look forward to them with an intensity of desire and expectation that will bring them together in gladness, make them spend the time in profit, and go away feeling it was good to be there ;—the result being, you will have what you never had—a live medical society, instead of one having a name to live while it is *dead.*

I know whereof I affirm. I was once a member of such medical body. It was immediately after the war of the States, the members were hungry for professional information. They had been almost entirely cut off from the usual sources of current medical knowledge for some years; had in fact been in a manner remitted to their own observation and experience. After the society had been in existence about a year, meeting weekly for most of the time, one of the members frankly confessed to having obtained more practical, working information from his fellows of the society and his study and preparation to meet them in discussion, than he acquired during two (2) courses of lectures he attended in the University of New York.

Now, gentlemen doctors, I am quite sensible here is a very pretty place to round to and stop; that I have said quite enough for the occasion; but I am tempted—and the temptation, as is too commonly the case with us, weak mortals, is too much for me,and I have a word more.

There is no one quality, more fundamentally important to professional men generally, (I might make the statement include all trades and avocations) but to members of the healing art especially, than the cultivation of what the French call the *esprit de corps*, professional pride, a disposition, as the apostle of the Gentiles termed it, to magnify mine (their) office; and a kindly, fraternal, sympathetic feeling for each other. I speak of a proper *esprit de corps* and kindly feeling of brotherhood as the same, for they are one and inseparable. Every professional man who loves his work for his works sake, loves his fellow-workers. No man ever made a doctor, whatever his opportunities or endowments, who practiced the healing art for the money in it. Such a one may get practice, may make money, but a doctor, never. Than the quality now under consideration there is nothing that I know of—

> "Aught that ever I could read,
> Could ever hear, by tale or history,"

that tends so directly to inspire a feeling of respect for the profession, to build it up in popular estimation and in the confidence of the community;—nothing that contributes so much to the comity and amenity of professional intercourse. I will not say there is no other means of cultivating this most desirable, indispensable, professional virtue, but I will say, there is no other equal to a properly conducted medical society, where the members, freed from all embarrasments and reservation, can fully and frankly express their convictions and exchange views one with the other. It at once, and forever, antidotes and stamps out that spirit of cold isolation and censoriousness from which spring envy, jealousy, detraction and the whole brood of vices and littleness that derogate so much from professional standing in popular estimation, and mar so sadly the intercourse of the members one with another. It is a great mistake to suppose that people in this world are not generally estimated at their true value. The complaint is sometimes heard that the healing art is not properly appreciated; its members not honored as they deserve. It is a mistake. No true medical man so feels. The healing art is appreciated; its members held at all they are worth. Show you deserve it, and the world will honor you; appreciate your high calling, and the world will appreciate it too; Esteem each other and the world will esteem you. Give, and it shall be given and you shall have in abundance. As in the domain of charity, so in this calling of ours: There is that giveth yet increasing, there is that withholdeth more than is meet, but tendereth to poverty. I had occasion some years ago to use these words, in a paper read before the Texas State Medical Association, and I beg to repeat them in your presence.

"That the physician, who is one in fact as well as in name, is entitled to consideration among his fellows, nobody, either in or out of the profession, doubts. That the members of the profession at large are not appreciated as the typical physician should be, is granted; but do these who bear the name, and who claim to share the honors of the profession, approach it in those elements of severe culture, scientific attainments and moral elevation which go to make up this distinguished character? In the development of sociological nomenclature, the medical is named a liberal profession, and its members are spoken of as belonging to the learned class. Is such classificaiion, such recognition, indicative of fact, or of usage of society that has become conventional and meaningless? That there are numbers, great numbers, of educated gentlemen in the profession in the United States who devote their lives to the acquisition of such knowledge as, in its practical application to the cure of disease, confers a benefit on their fellows, the value of which cannot be over-estimated, is a fact which should cause professional pride and congratulation. But it is no less true that crowds are rushing into this high and most delicate calling as the unthinking horse into battle, with no preparation at all adequate for the discharge of its arduous duties, and which preparation is indispensable to meet its profound obligations. Many, indeed, rely for such success, as their ignoble aspirations lead them to look forward to, upon the cultivation and excercise of such bearing to society as is calculated at once to degrade the profession and to debauch public sentiment. Conscious of no worth, knowing themselves to be mere animal fungi, parasites upon the body social—*fruges consumere nati*—mere consumers of the public bounty, contributors in no sense to the public weal, and having, therefore, forfeited their own self-respect, as every such man must, is it reasonable for them to claim the respect of others?

When looking upon one of these self-important creatures, taking upon himself the responsibilities that necessarily attach to the character of the physician, and claiming at the hands of society the consideration due to such a character, with none of the cultivation required to meet the one, nor any of the elements of true manhood to support the claims of the other, one is tempted to call down upon the miscreants the old anathema: "Wherever fire burns or water runs; wherever ships float or land is tilled; wherever the skies vault themselves, or the lark carols to the dawn, or sun shines and grass grows in his rays; wherever God is worshiped in temples or heard in thunder; wherever man is honored or woman loved—therefrom, henceforth and forever, let there be to him no part or lot in the honor of man or the love of woman. Let Ixion's revolving wheel, the overmantling cup at which Tantalus might not quench his unquenchable thirst, the insatiate vulture gnawing at the immortal heart of Prometheus, the rebel giants, writhing in the volcanic fires of Etna—be but faint types of his doom."

